# Editorial: Endocrine dysfunctions and immunometabolic pathways in autoimmune-related cancers

**DOI:** 10.3389/fendo.2026.1809287

**Published:** 2026-02-27

**Authors:** Tao Zhang

**Affiliations:** Cancer Center, West China Hospital, Sichuan University, Chengdu, China

**Keywords:** autoimmune, cancer, endocrine, immunotherapy, metabolism

The pathogenesis of cancer is increasingly viewed as a process driven by both systemic and local microenvironmental disturbances (1). The interplay between endocrine homeostasis, immune surveillance, and cellular metabolism forms a core regulatory network in this process (2, 3). Specifically, endocrine disorders characterized by dysregulated hormone signaling and autoimmune conditions marked by chronic immune activation can synergistically remodel the tumor microenvironment through shared immunometabolic pathways, thereby influencing cancer susceptibility, progression, and therapeutic response (4–6). This Research Topic, “Endocrine Dysfunctions and Immunometabolic Pathways in Autoimmune-Related Cancers,” aims to systematically dissect this complex network of interactions. It brings together six studies spanning clinical observations, mechanistic explorations, and therapeutic assessments. Collectively, these works elucidate how endocrine-immune crosstalk shapes specific cancer phenotypes—from risk definition and rare clinical presentations to the management of treatment-related complications—while revealing novel metabolic targets embedded within this network.

First, the study by Sun et al. investigates the rare clinical co-occurrence of medullary and papillary thyroid carcinoma. Their comparative analysis indicates that patients with both tumor types exhibit disease invasiveness and prognosis similar to those with medullary thyroid carcinoma alone. This finding suggests potentially independent oncogenic drivers. However, it also raises a profound question: Could a permissive local niche, shaped by common endocrine or immune factors, provide the foundation for the development of both tumors within the same gland? This question connects the case study to broader theories of the tumor microenvironment.

The review by Wang et al. systematically elaborates on the multifaceted functions of adipose-derived mesenchymal stem cells (ADSCs), positioning them as central metabolic and immunomodulatory integrators within the tumor microenvironment. This review clarifies that ADSCs can translate systemic endocrine and metabolic signals into highly context-dependent pro- or anti-tumor effects. This work highlights the plasticity of stromal cells as a critical bridge connecting systemic metabolic disorders to local tumor progression, clearly exemplifying the central role of the immunometabolic axis in cancer advancement.

The work by Xu et al. employs a bioinformatics approach to establish the prognostic significance of bile acid metabolism (BAM) in hepatocellular carcinoma (HCC), identifying *NPC1* as a key biomarker and oncogenic driver. This study directly links this core hepatic metabolic pathway to tumor aggressive behavior, providing not only correlative evidence but also proposing a potential metabolic axis for risk stratification and intervention.

The pharmacovigilance assessment by Brown et al. rigorously analyzes lymphoma risk in patients with lipodystrophy, a severe metabolic disorder involving leptin deficiency. By reporting cases in both drug-treated and treatment-naïve patients, this analysis crucially indicates that the inherent metabolic and immune dysregulation of the disease itself constitutes a significant carcinogenic risk factor. This distinction is fundamental for the precise risk-benefit assessment of therapeutic strategies in rare diseases.

The detailed case report by Zhao et al. describes a case of severe thyroiditis induced by sintilimab. This report vividly embodies the direct clinical collision between oncology and endocrinology, where potent anti-cancer immunotherapy can trigger severe autoimmune-like endocrine toxicity. It underscores the shared immunological basis for both tumor eradication and endocrine tissue damage, emphasizing the necessity of adhering to relevant clinical guidelines for vigilant monitoring and early management.

Finally, the study by Shen et al. explores the role of fatty acid metabolism (FAM) in papillary thyroid cancer (PTC), constructing a prognostic signature centered on *SCD*. Their work demonstrates that intrinsic metabolic reprogramming in tumor cells not only directly drives proliferation and invasion but also shapes an immunosuppressive microenvironment. This links metabolic dysregulation to both the tumor biology of PTC and its mechanisms of immune evasion.

In summary, the research in this Topic presents a multidimensional understanding of the endocrine-immune-cancer axis. Several common themes emerge: the prognostic and therapeutic relevance of specific metabolic pathways; the dual nature of immune modulation in controlling cancer versus inducing endocrine pathology; and the critical importance of distinguishing disease-inherent risk from treatment-associated effects in clinical evaluation. Furthermore, the diversity of contexts involved—from common thyroid and liver cancers to rare lipodystrophy and immunotherapy toxicity—demonstrates the broad relevance of these interactive mechanisms, advocating for an integrated research paradigm that transcends traditional disciplinary boundaries (7–9).

These findings collectively point to several key directions for future research: First, targeting metabolic nodes such as SCD or NPC1 to disrupt tumor growth and remodel the immune microenvironment. Second, developing predictive biomarkers that combine metabolic and immune features for cancer risk stratification in populations with autoimmune/endocrine disorders and for predicting immunotherapy toxicity. Third, designing mechanism-based combination strategies, such as integrating metabolic modulators with immunotherapy. Fourth, implementing personalized monitoring and management plans for patients at this complex intersection.

In conclusion, the contributions to this Research Topic mark significant progress in deciphering the intricate links between endocrine health, immune function, and cancer metabolism. By integrating clinical insights with molecular mechanisms, they collectively advance the framework for more precise prevention, prognostication, and treatment strategies for autoimmune-related and endocrine-influenced cancers.
